# Unrecognized severe acute respiratory coronavirus virus 2 (SARS-CoV-2) seroprevalence among healthcare personnel in a low-prevalence area

**DOI:** 10.1017/ice.2020.1341

**Published:** 2020-11-20

**Authors:** Vishal P. Shah, Caitlin M. Hainy, Melanie D. Swift, Laura E. Breeher, Elitza S. Theel, Priya Sampathkumar

**Affiliations:** 1 Division of Preventive, Occupational, and Aerospace Medicine, Mayo Clinic, Rochester, Minnesota; 2 Occupational Health Services, Mayo Clinic, Rochester, Minnesota; 3 Department of Laboratory Medicine and Pathology, Division of Clinical Microbiology, Mayo Clinic, Rochester, Minnesota; 4 Division of Infectious Diseases, Mayo Clinic, Rochester, Minnesota

*To the Editor—*Healthcare personnel (HCP) caring for patients with severe acute respiratory syndrome coronavirus 2 (SARS-CoV-2) may have higher rates of coronavirus disease 2019 (COVID-19) than other HCP. A study of asymptomatic HCP in Texas found much higher rates of SARS-CoV-2 reverse-transcriptase PCR (RT-PCR) positivity in COVID-19–facing HCP (4.8%) compared to other HCP (0.6%).^1^ However, RT-PCR evaluation alone may lead to an underestimation of COVID-19 infections due to testing only symptomatic cases, timing of sample collection, and/or false-negative tests.^2^ Antibody assessment could provide further insight into the prevalence of COVID-19 among HCP. Immunoglobulin G (IgG) antibodies to SARS-CoV-2 typically develop within 8–14 days of symptom onset, and most are seropositive by 21 days.^3^ A multistate study of frontline HCP revealed significant heterogeneity in seroprevalence, ranging from 0.8% to 31.2%; however, HCP who tested positive by RT-PCR were also included.^4^ Our study sought to determine the seroprevalence of HCP who did not previously test positive for COVID-19 by RT-PCR at a tertiary-care medical center in the midwestern United States.

Mayo Clinic employees in Rochester, Minnesota, were recruited between May 25 and July 9, 2020, and were placed into high- and low-risk cohorts based on their role and work unit. The high-risk cohort included HCP in a direct patient care role, working in the emergency department, COVID-19 intensive care unit, or COVID-19 general care ward. Low-risk HCP were those not involved in direct patient care. HCP who previously tested positive or who had a household member previously test positive for COVID-19 were excluded from the study. All study participants completed a questionnaire about occupational and community exposure and were asked about symptoms consistent with COVID-19. This study was approved by the Mayo Clinic Institutional Review Board (no. 20-003787).

All enrolled HCP were first screened using the Euroimmun anti–SARS-CoV-2 IgG ELISA (Lubeck, Germany).^5^ Positive or indeterminate samples were retested using either the Roche Diagnostics anti-SARS-CoV-2 Total Antibody Immunoassay (Roche Diagnostics, Rotkreuz, Switzerland) or the Ortho-Clinical Diagnostics anti–SARS-CoV-2 IgG Antibody Immunoassay (Ortho-Clinical Diagnostics, Raritan, NJ). Only participants with positive tests by 2 assays were considered seropositive for antibodies to SARS-CoV-2. RT-PCR testing records were assessed through occupational health records from March 9 through June 30, 2020. The χ^2^ and Fisher exact tests were used for the statistical analysis.

In total, 586 participants were enrolled, and 568 completed the survey and laboratory analysis (Table 1). Overall, 2 of 320 (0.63%) HCP in the high-risk cohort tested positive for IgG antibodies against SARS-CoV-2, while 0 of 248 (0%) tested positive in the low-risk cohort (OR, 3.90; *P* = .51). The 2 individuals with antibodies to SARS-CoV-2 did not report breaches in personal protective equipment (PPE), nor were they advised to quarantine due to a known exposure to a person with COVID-19. One seropositive study participant reported symptoms of chills, myalgias, diarrhea, and a headache, and the second participant reported a headache. A higher number of HCP in the high-risk cohort were placed on a quarantine due to known exposure to a person with COVID-19 compared to the low-risk cohort (odds ratio [OR], 12.1; *P* = .0016).

**Table 1.** Demographic Information and Survey Analysis

Characteristic	High-Risk Cohort (n=320)	Low-Risk Cohort (n=248)	*P* Value^a^
Age, mean y (SD)	36.3 (10.8)	47.1 (11.7)	**<.001**
Sex, no. (%)			
Female	250 (78.1)	217 (87.5)	**.004**
Male	70 (21.9)	31 (12.5)	…
Ethnicity, no. (%)			
Non-Hispanic/White	304 (95)	240 (96.8)	.401
Hispanic or Latinx	10 (3.1)	3 (1.2)	.163
Unknown/Not reported	6 (1.9)	5 (2)	1
Role, no. (%)			
Nurse	223 (69.7)	6 (2.4)	**<.001**
Nurse practitioner/Physician assistant	18 (5.6)	…	**<.001**
Patient care assistant	19 (5.9)	…	**<.001**
Physician	42 (13.1)	…	**<.001**
Respiratory Therapist	17 (5.3)	…	**<.001**
Medical/Administrative assistant	…	129 (52)	**<.001**
Research personnel	…	51 (20.6)	**<.001**
Other	…	62 (25)	**<.001**
Provided care to a patient who tested positive for COVID-19?			
Yes, no. (%)	288 (90.3)	0 (0)	…
No, no (%)	8 (2.5)	248 (100)	…
Unsure	23 (7.2)	0 (0)	…
Reported any symptoms since March 1, 2020, no. (%)			
None	212 (66.3)	179 (72.2)	.144
Fever	12 (3.8)	5 (2)	.322
New cough	19 (5.9)	17 (6.9)	.729
Shortness of breath	7 (2.2)	11 (4.4)	.151
Chills	18 (5.6)	7 (2.8)	.148
Repeated shaking	2 (0.6)	2 (0.8)	1
Muscle aches	25 (7.8)	13 (5.2)	.241
Sore throat	36 (11.3)	24 (9.7)	.584
Diarrhea	19 (5.9)	25 (10.1)	.081
Headache	66 (20.6)	44 (17.7)	.454
Partial or complete loss of sense of smell	2 (0.6)	3 (1.2)	.658
Did you report a PPE Breach during the care of a patient with COVID-19?			
No	244 (84.7)	…	…
Yes	31 (10.8)	…	…
Unsure	13 (4.5)	…	…
Since March 1, 2020, have you been advised to quarantine due to an exposure to a person with confirmed COVID-19?			
No	305 (95.3)	246 (99.2)	**.006**
Yes	15 (4.7)	1 (0.4)	**.002**
Unsure	0 (0)	1 (0.4)	.437

Note. SD, standard deviation.

a Student *t* test was used for continuous variables and the Fisher exact test was used for categorical variables.

Based on role and employment location, there were 1,348 employees who would have been eligible to enroll in the high-risk cohort. From March 9 to June 30, 2020, 7 of these employees tested positive by RT-PCR, 3 of whom had known community exposures. The RT-PCR positive rate between the high-risk cohort (0.52%) and the non–high-risk cohort (0.57%) were similar and were also comparable to overall RT-PCR prevalence rates in the region (0.67% as of July 1, 2020).^6^

Previous data describing COVID-19 infection rates in HCP have been mixed. The multistate study of frontline HCP and other studies have shown significant heterogeneity among frontline HCP that generally correlated with community rates.^4^ A study evaluating IgG antibodies to SARS-CoV-2 among HCP in Germany found the overall rate of unrecognized prior infection to be 1.6%.^7^ However, the use of single antibody assays in low-prevalence areas may lead to high rates of false-positive results. A Centers for Disease Control and Prevention (CDC) study assessing antibody prevalence across 10 sites in the United States from March 23 to May 12, 2020, revealed highly variable prevalence rates ranging from 1% to 6.9%.^8^ An orthogonal testing algorithm was utilized; however, the study did not specify whether individuals had been symptomatic or previously tested by RT-PCR.

Here, we present serology and RT-PCR data to determine the prevalence of recognized and unrecognized COVID-19 infections among HCP. In this low-prevalence setting, HCP in the high-risk cohort are much more likely to encounter persons with COVID-19 in the occupational setting compared to the community. Despite providing care to patients with COVID-19 and having higher odds of being quarantined due to an exposure to a person with COVID-19, the rate of SARS-CoV-2 infection based on combined seroconversion and RT-PCR positivity did not differ between high- and low-risk HCP, and they were similar to community rates.

Our study has several limitations. Given the kinetics of antibody development against SARS-CoV-2, individuals tested shortly after infection may not have mounted an antibody response. Additionally, the county in which the hospital is situated also had a low prevalence of COVID-19, as determined by the percentage of positive by RT-PCR tests.

Overall, HCP regularly caring for patients with COVID-19 did not have significantly higher rates of COVID-19 infection compared to other HCP at a tertiary-care center in Minnesota. In particular, rates of unrecognized infection were low. These data support the efficacy of current processes to identify and isolate COVID-19 patients and to limit HCP exposure to COVID-19 through administrative practices, training, and rigorous use of PPE.
